# Conserve primers for sequencing complete ungulate mitochondrial cytochrome c oxidase I (COI) gene from problematic and decomposed biological samples

**DOI:** 10.1080/23802359.2016.1247672

**Published:** 2017-02-06

**Authors:** Ajit Kumar, Mirza Ghazanfar Ullah Ghazi, Bhim Singh, Syed Ainul Hussain, Dinesh Bhatt, Sandeep Kumar Gupta

**Affiliations:** a Department of Animal Ecology and Conservation Biology, Wildlife Institute of India, Dehradun, India;; b Department of Zoology and Environmental Science, Gurukula Kangari University, Haridwar, India;; c Department of Landscape Level Planning & Management, Wildlife Institute of India, Dehradun, India

**Keywords:** Mitochondrial cytochrome c oxidase I gene, non-invasive and decomposed samples, PCR amplification, ungulate species

## Abstract

We describe six novel ungulate-specific conserved primers for sequencing the complete mitochondrial cytochrome c oxidase I (COI) gene of selected threatened species from degraded samples for effective conservation planning. These primers amplified 301–1599 bp DNA fragments in various combinations. The method described may assist in the sequencing of either complete gene from moderate to good quality DNA or shorter fragment from degraded DNA.

## Introduction

Terrestrial mammals are threatened due to extinction risk, and around 50% of them are showing a declining trend (Channell & Lomolino 2000; Ceballos et al. 2005). Among these, the South Asian mammals are the most endangered group (Schipper et al. 2008), specially the ungulates, which are declining due to environmental changes, impacts of anthropogenic pressure on wildlife habitats and poaching (Estes et al. 2006). Conservation success largely depends upon identifying vulnerable species and understanding the environmental factors that support their persistence in human-dominated landscapes. In recent years, molecular taxonomy has helped in resolving the phylogeny of cervids (Pitra et al. 2004; Gilbert et al. 2006) resulting in clarity on species distribution and relatedness for effective conservation planning. However, studies indicate further revision in the molecular phylogeny (Groves & Grubb 2011; Timmins et al. 2015). Successful conservation efforts depend upon the identification of evolutionary significant units (ESU) of vulnerable species. The mitochondrial DNA (mtDNA) cytochrome c oxidase I (COI) gene has been widely used for biological identification and phylogenetic studies (Weibel & Moore 2002; Hebert et al. 2003). Further enriching of its database with the DNA sequences of COI gene would be an asset for the identification of ESU of vulnerable species exhibiting declining trend and reduced genetic variations. It requires large amounts of sequence data from different populations.

With increasingly stringent wildlife laws (e.g. Indian Wildlife (Protection) Act, 1972), obtaining permission for collection of invasive biological samples is difficult. Therefore, researchers often utilize decomposed and non-invasively collected biological samples for addressing the genetic issue. Amplification and sequencing of a complete gene from the DNA extracted from such samples is a challenging task. With our experience of working on population genetics and molecular phylogeny of swamp deer (*Rucervus duvaucelii*), sambar (*Rusa unicolor*), hog deer (*Axis porcinus*), wild pig (*Sus scrofa*) and other ungulates (Gupta et al. 2013a; Angom et al. 2015), we describe novel primers for sequencing the complete mtDNA COI gene by amplification of shorter fragment from the DNA extracted from non-invasive and degraded samples.

Complete COI sequences with the flanking region of various ungulate species were obtained from GenBank and aligned using ClustalW (Thompson et al. 1994). After alignment of the sequences, the homologous regions were selected for designing three pairs of conserved primer (Table 1).

**Table 1.** Six primers designed for amplification of either complete or partial fragment of mtDNA COI gene.

S. No.	Primer name	Sequence (5′→3′)
1	COI (5298-323) F1	TACAGTCTAATGCTTCACTCAGCCA
2	COI (5577-599) F2	GTACCGCTAATAATTGGTGCTCC
3	COI (5599-571) R1	GGGGCAATTATTAGGGGAACTAGTCA
4	COI (6024-046) F3	CAACACTTGTTCTGATTCTTCGG
5	COI (6052-027) R2	GGGTGGCCAAAGAATCAGAACAAGTG
6	COI (6897-876) R3	GGGGGTTCGATTCCTTCCTTTC

Primer positions were derived from the complete mtDNA sequence of Rusa unicolor swinhoei (EF035448).

**Figure 1.**
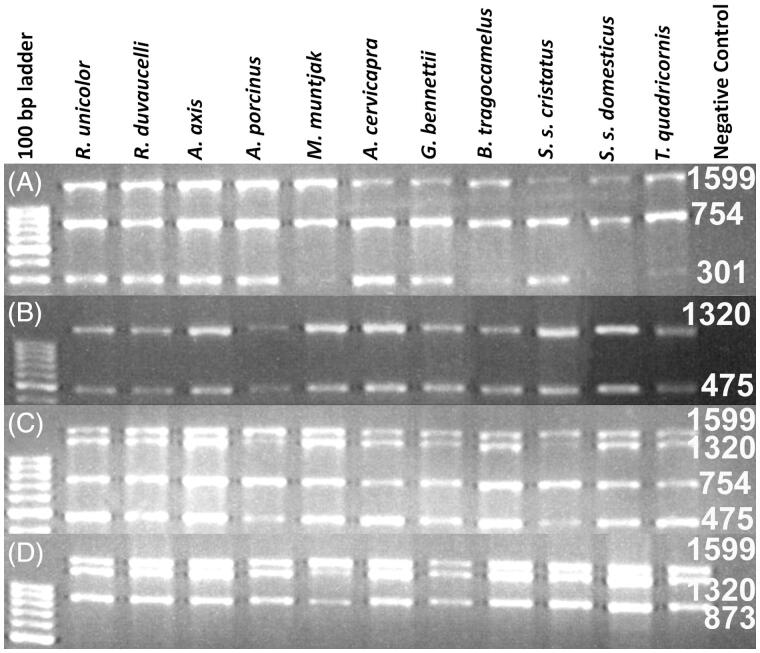
Image of the agarose gel showing amplification result of multiplex PCR reaction with the DNA extracted from partially decomposed tissue of a verity of ungulate species as labelled at each lane. (A) PCR amplification with F1, R1, R2 and R3 primers; (B) amplification with F1, F2, R2 and R3 primers; (C) amplification with F1, F2, R2 and R3 and (D) amplification with F1, F2, F3 and R3. Numeric are the base pair size of respective amplicons.

We used biological samples collected from the field for validation of primers. DNA was extracted (Gupta et al. 2013b) and subjected to PCR amplification using different primer combinations (Table 2). A range of annealing temperature was tested for multiplex amplification, and optimum amplification was obtained at 56 °C. PCR amplification was carried out in a 20 μl reaction volume containing 1 μl of the extracted DNA, 4 pmol of each primer and 1× Multiplex PCR Kit (Qiagen, Hilden, Germany). We performed PCR in the following steps: initial denaturation at 95 °C for 15 min, followed by 35 cycles of denaturation at 95 °C for 45 seconds, annealing at 56 °C for 1 min, and extension at 72 °C for 1.5 min. Final extension was at 72 °C for 10 min. We used 2.2% agarose gel containing ethidium bromide to visualize PCR products under a UV transilluminator (Figure 1). These were sequenced from both the strands using ABI 3130 Genetic Analyser. Sequences were aligned using SeqScape v2.5 (Applied Biosystems, Foster City, CA). A neighbour-joining phylogenetic tree was constructed using the Kimura 2-parameter method (Kimura 1980) with MEGA 7 (Kumar et al. 2016).

**Table 2.** All possible combinations of the primers during PCR amplification with tested samples and expected amplicon lengths.

S. No.	Primer combination	Expected amplicon length(s)	Sample type
1	F1 and R1	301 bp	T/B/H/A/Bo/F
2	F1 and R2	754 bp	T/B/H/A/Bo
3	F1 and R3	1599 bp	T/B
4	F2 and R2	475 bp	T/B/H/A/Bo/F
5	F2 and R3	1320 bp	T/B
6	F3 and R3	873 bp	T/B/H/A
In multiplex reactions			
7	F1, R1, R2 and R3	301, 754 and 1599 bp	
8	F2, R2 and R3	475 and 1320 bp	
9	F1, F2, R2 and R3	475, 754, 1320 and 1599 bp	
10	F1, F2, F3 and R3	873, 1320 and 1599 bp	

Where T, B, H, A, Bo and F stand for fresh and partially decomposed tissue, blood, hairs, antlers, bone and faecal samples respectively.

## Results and conclusions

**Figure 2.**
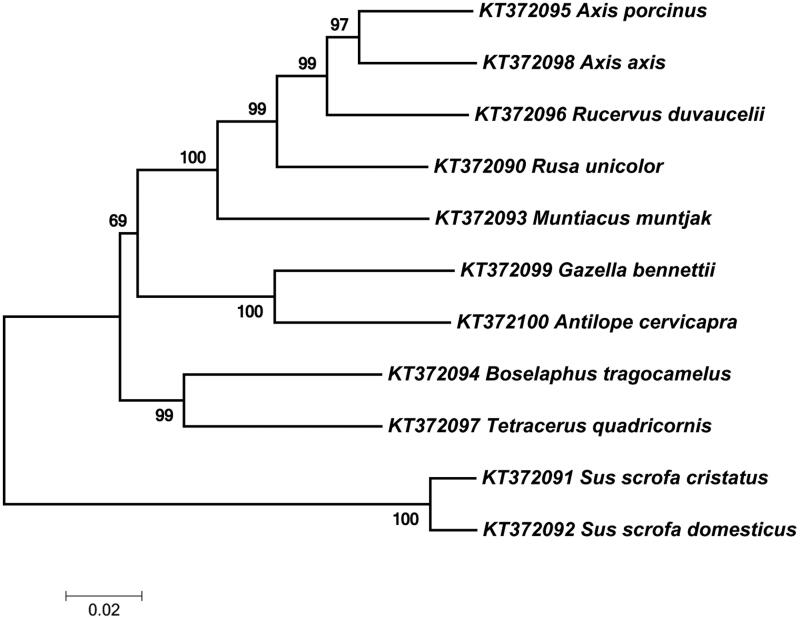
Phylogenetic tree based on neighbour-joining method using MEGA 7. The percentage of replicate trees in which the associated taxa clustered together in the bootstrap test (1000 replicates) are shown next to the branches.

Expected PCR amplicons were obtained with different primer combinations (Table 2 and Figure 1). Less than 1 KB fragments (301, 475, 754, and 837 bp) were amplified with the DNA extracted from all the biological samples excluding faecal samples (Table 2). The maximum amplicon size from the faecal DNA was 475 bp. PCR products of 1320 and 1599 bp were amplified from the DNA extracted from fresh and less decomposed tissue samples (Table 2). A complete gene consensus sequence was obtained for various tested ungulate species and deposited in the NCBI GenBank (KT372090-2100), as shown in Table 3. The neighbour-joining (NJ) tree exhibited the expected phylogenetic relationship among the tested species (Figure 2).

**Table 3.** List of ungulate species used for validation of primers.

S. No.	Amplification and sequencing with ungulate species	NCBI accession No.
1	Sambar (*Rusa unicolor*)	KT372090
2	Indian wild pig (*Sus scrofa*)	KT372091
3	Domestic pig (*Sus scrofa*)	KT372092
4	Barking deer (*Muntiacus muntjak*)	KT372093
5	Nilgai (*Boselaphus tragocamelus*)	KT372094
6	Hog deer (*Axis porcinus*)	KT372095
7	Swamp deer (*Rucervus duvaucelii*)	KT372096
8	Four-horned antelope (*Tetracerus quadricornis*)	KT372097
9	Chital (*Axis axis*)	KT372098
10	Chinkara (*Gazella bennettii*)	KT372099
11	Blackbuck (*Antelope cervicapra*)	KT372100

Alphanumeric are the NCBI accession numbers of our sequences.

Availability of virtue of DNA sequences from a different population of species facilitates the improved phylogenetics. For the collection of invasive samples, immobilization of wild animal using tranquilization drugs involves risk on their life. However, tranquilization-free collection of samples from the endangered wild animal is an efficient way of increasing the sample size. Therefore, amplification of smaller fragments to generate a complete gene sequence is a convenient way of enhancing databases for those species when invasive samples are difficult to obtain. The availability of complete sequences for multiple genes from a variety of species can improve the reliability of sequence-based species identification and phylogeny. The primer combinations described here will be beneficial for amplification and sequencing of complete COI gene of ungulate species using decomposed biological samples. The COI sequence along with widely deployed cytochrome *b* gene can be used to generate the unambiguous phylogenic tree.
